# Rising trends in iatrogenic urogenital fistula: A new challenge

**DOI:** 10.1002/ijgo.13037

**Published:** 2020-01-13

**Authors:** Nasira Tasnim, Kauser Bangash, Oreekha Amin, Sobia Luqman, Hadia Hina

**Affiliations:** ^1^ Department of Maternal and Child Health Pakistan Institute of Medical Sciences Islamabad Pakistan

**Keywords:** Iatrogenic fistula, Obstetric fistula, Pakistan, Trends

## Abstract

**Objective:**

To analyze trends in iatrogenic urogenital fistula among patients admitted for fistula repair at the Pakistan Institute of Medical Sciences, Islamabad.

**Methods:**

In this longitudinal study, all patients who presented for fistula repair between 2006 and 2018 were included in the study. Patient data were collected on age, parity, and type and etiology of fistula, which was classified as ischemic or iatrogenic.

**Results:**

Of 634 fistula patients, 371 (58.5%) had iatrogenic fistula, while 263 (41.5%) patients developed ischemic fistula due to obstructed labor. Mean age of patients was 31.6 years. Yearly trends showed an increase in iatrogenic fistula from 43.2% in 2006–2008 to 71.4% in 2017–2018. The major etiological contributor to iatrogenic fistula was hysterectomy (52.5%), followed by cesarean hysterectomy (26.4%), and cesarean delivery (19.9%).

**Conclusion:**

A rising trend in iatrogenic fistula was observed. This emphasizes the need for optimization of surgical approaches and surgical skills. Moreover, gynecologic surgeries should be restricted to authorized gynecologic surgeons.

## Abstract 2

A rising yearly trend in iatrogenic fistula was observed over a 12‐year period in Pakistan.

## 1 INTRODUCTION

Urogenital fistula is a distressing and socially debilitating health condition for women. It is estimated that approximately 30 000–130 000 new cases occur annually worldwide.^1^ In Pakistan, an estimated 3500 cases of obstetric fistula occur every year.^2^ Furthermore, the prevalence of urogenital fistula in women of reproductive age has been reported as 1.60 per 1000 women in South Asia.^3^ Determining an accurate prevalence of fistula is still difficult owing to inadequate reporting and lack of disclosure of the condition by those affected.^4^

It has been reported that low‐resource countries have a higher incidence of obstetric ischemic fistula compared with high‐resource nations.^5^ However, contrary to the previous trend, a significant shift has been observed regarding the etiology of female urogenital fistula. Recent studies have shown a growing incidence of iatrogenic fistula.^6^, ^7^ Trends observed in various studies conducted over a period of 13 years in Pakistan show a significant increase in iatrogenic fistula.

The incidence of ischemic fistula in Pakistan declined from 70%–80% in 2004–2005^8^, ^9^, ^10^ to 45%–56% in 2015–2016).^11^, ^12^ This changing trend could be attributed to improvement in maternity skills and referral systems over time. As reported by the joint UNICEF/WHO database, in Pakistan, 38.8% of deliveries were carried out by skilled birth attendants (doctor, nurse, midwife, lady health worker) in 2006–2007, while in 2017–2018, 69% were attended by skilled birth attendants.^13^ However, the increasing rate of obstetric interventions and gynecologic pelvic surgeries performed by personnel lacking the appropriate skills may be the cause of the rising rate of iatrogenic fistula.

Although a significant decrease in obstetric ischemic fistula has been observed, the remaining obstetric fistula cases reflect poor access to and inadequate obstetric services in remote areas. On the contrary, iatrogenic fistula reflects suboptimal surgical techniques that could be prevented through optimization and refinement of surgical skills.

Previous studies in Pakistan were conducted in different settings and in different years. The aim of the present study was to analyze the changing trends in the frequency of iatrogenic fistula injuries among patients admitted for fistula repair at a single regional fistula center, the Pakistan Institute of Medical Sciences (PIMS) Islamabad, based on almost 12 years of audit data.

## 2 MATERIALS AND METHODS

This longitudinal study of prospectively collected data was conducted at the regional fistula center of the Pakistan Institute of Medical Sciences, Islamabad. The study included all patients diagnosed with urogenital fistula who underwent surgical repair between November 2006 and June 2018. Patient data were collected on age, parity, and type and etiology of fistula.

All patients underwent examination under anesthesia and intravenous urography to localize and classify the type of fistula. Urogenital fistula was classified as iatrogenic if the fistula developed after pelvic surgery (hysterectomy for nonobstetric causes), cesarean delivery, or cesarean hysterectomy. Ischemic fistula was classified as all low‐level fistulae (vesicovaginal, urethral; procuring necrotic tissue) that developed after obstructed labor of more than 24 hours followed by vaginal/instrumental delivery or cesarean delivery.

Ethical approval was obtained from the Pakistan Institute of Medical Sciences institutional review board. The study was discussed with recruited patients and informed consent was provided.

## 3 RESULTS

A total of 634 patients underwent urogenital fistula repair over the study period and were included. The mean age of patients was 31.6 ± 10.26 years (range, 15–70 years). Of the total, 371 (58.5%) were classified as iatrogenic fistula, whereas 263 (41.5%) were ischemic fistula. Among those with iatrogenic fistula, 195 (52.5%) patients developed fistula after hysterectomy performed for nonobstetric causes, 98 (26.4%) after cesarean hysterectomy, 74 (19.9%) after cesarean delivery (for obstetric reasons other than obstructed labor), and 4 (1.1%) after dilation and curettage. For patients with ischemic fistula, the causes were associated with obstructed vaginal delivery in 144 (54.7%), cesarean delivery in 99 (37.6%), and instrumental delivery in 20 (7.6%) patients (Table 1).

**Table 1:** Occurrence and cause of iatrogenic and ischemic fistula among study participants (n=634), 2006–2018

Fistula type and cause	No. (%)
Iatrogenic fistula (n=371)	
Hysterectomy (nonobstetric)	195 (52.5)
Cesarean hysterectomy	98 (26.4)
Cesarean delivery	74 (19.9)
Dilation and curettage	4 (1.1)
Ischemic fistula (n=263)	
Vaginal delivery	144 (54.7)
Cesarean delivery	99 (37.6)
Instrumental delivery	20 (7.6)

**Figure 1:**
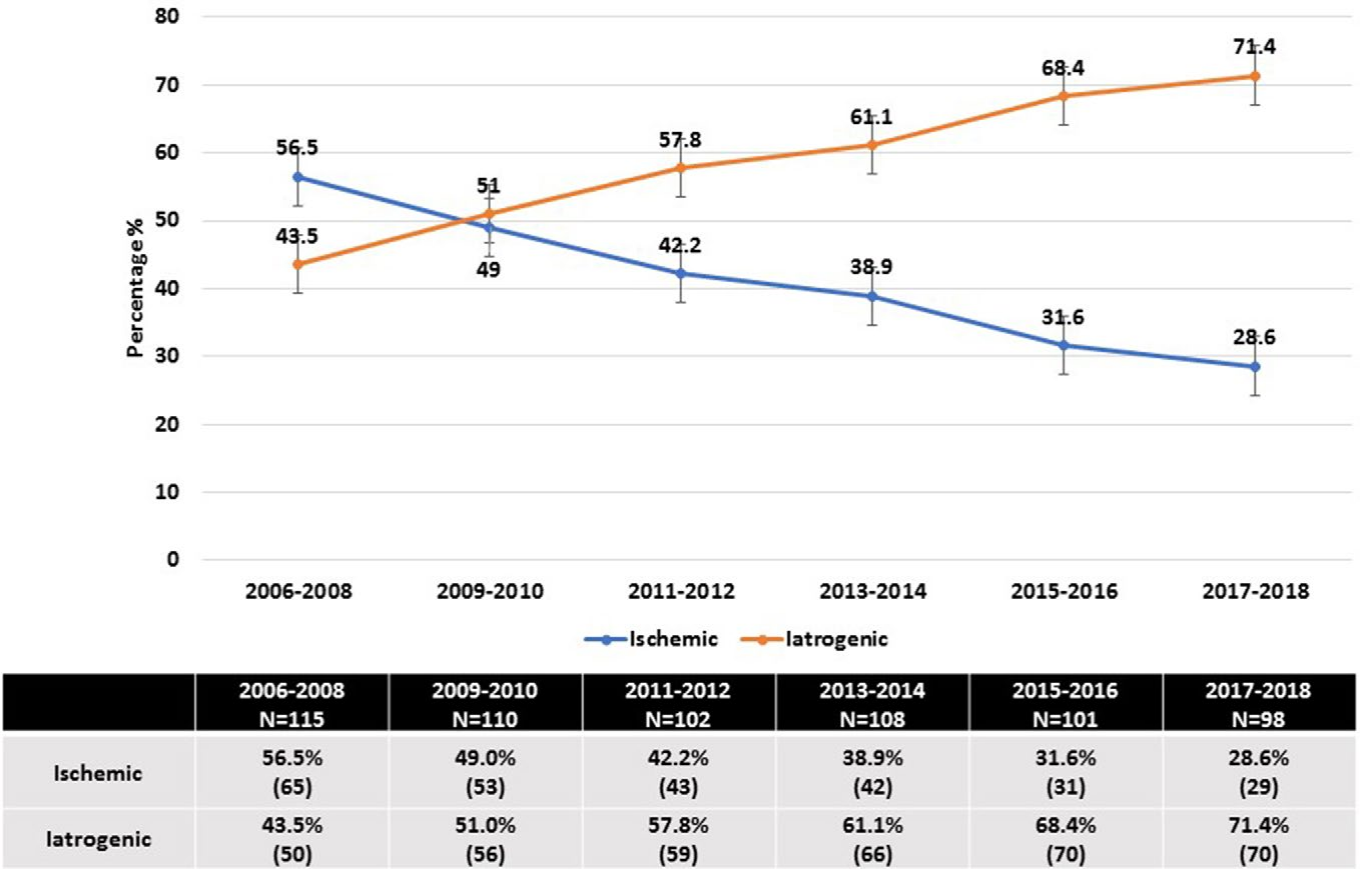
Trends in iatrogenic and ischemic fistula, 2006–2018.

A rising trend in iatrogenic fistula was found over the study period (2006–2018) from 43.5% to 71.4%. A decreasing trend in ischemic fistula, from 56.5% to 28.6%, was also observed (Fig. 1).

**Figure 2:**
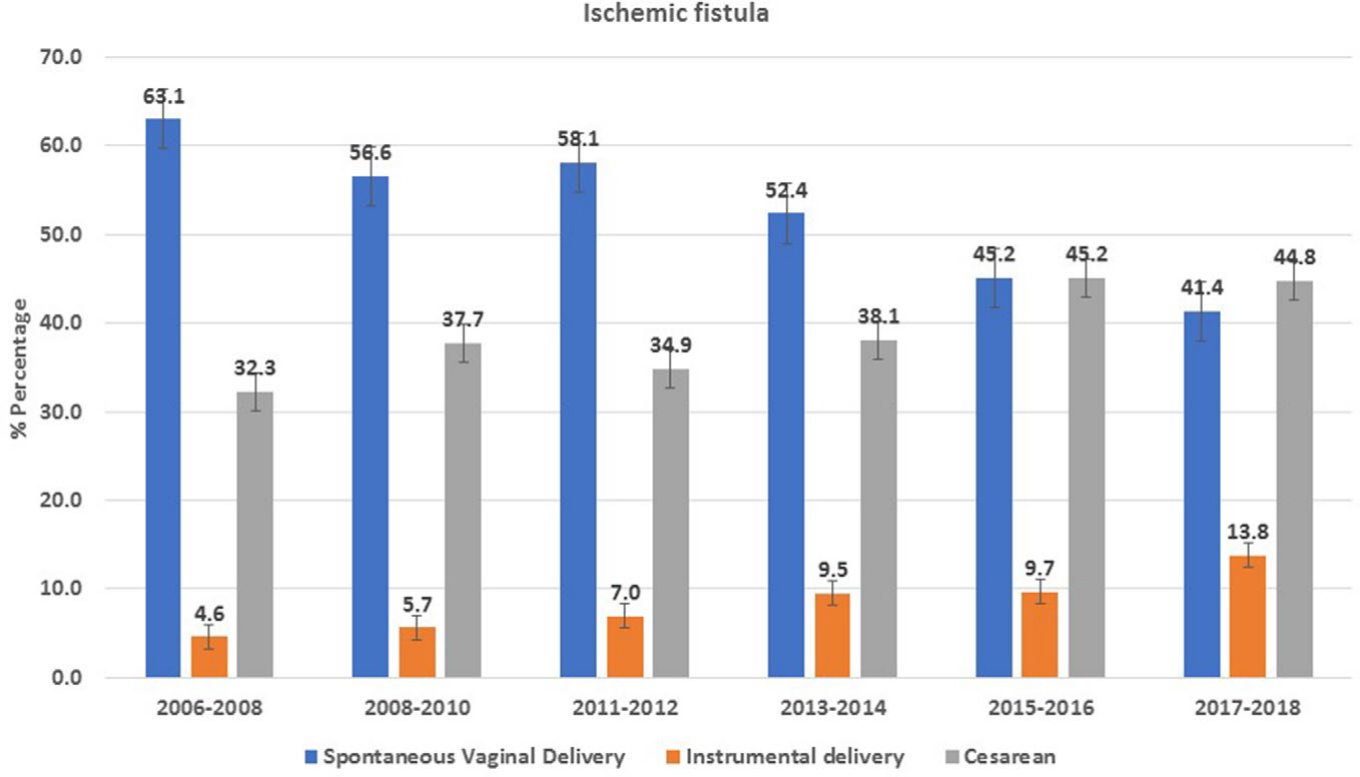
Trends in etiological factors for ischemic (obstetric) fistula, 2006–2018.

**Figure 3:**
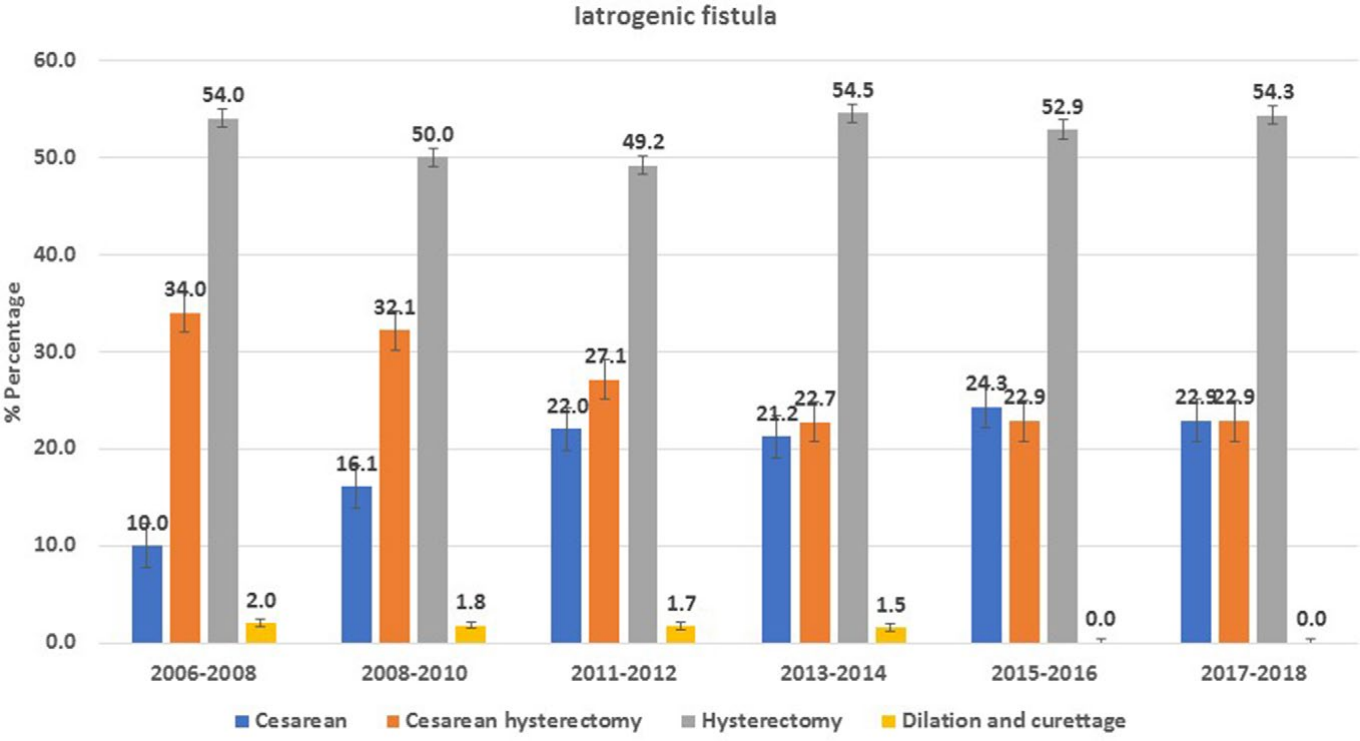
Trends in etiological factors for iatrogenic fistula, 2006–2018.

In terms of yearly trend, hysterectomy was the major etiological contributor to iatrogenic fistula, followed by cesarean delivery (Fig. 2). However, cesarean delivery as the cause of fistula showed a continuous rising trend over the years. Furthermore, curettage of the uterine cavity was found to be a minor contributor to iatrogenic fistula. A declining trend in ischemic fistula developing after obstructed vaginal delivery was observed (Fig. 3).

## 4 DISCUSSION

In the past, the causes of urogenital fistula in low‐resource countries have not been linked to iatrogenic origins. However, cases of iatrogenic fistula appear to be increasing in recent years. A retrospective study conducted in 11 countries, mostly in Africa, between 1994 and 2012 reported that 13.2% of urogenital fistula cases were caused by iatrogenic errors.^7^ A study from Ethiopia (2011–2015) reported that among 2500 fistula cases, 24.6% were attributed to surgical cause, particularly cesarean delivery and hysterectomy.^14^ An Indian study conducted from 2007 to 2013 reported that 39% of genitourinary fistulae were iatrogenic.^15^ A further study, conducted from 2011 to 2015 in India, reported that 65% of fistula cases were iatrogenic.^16^ Similarly, urogenital fistula studies conducted in Pakistan over various years have shown a gradual rising trend in iatrogenic fistula. One study conducted in 2005 reported that 75% of fistulae were caused by obstructed labor compared with 28.6% that were iatrogenic.^9^ Finally, a 2015 study reported that 53.6% of fistula cases were caused by obstructed labor versus 39% that had iatrogenic causes.^11^

These previous studies were conducted in different settings over different time periods. Over almost 12 years in the present study, the rate of iatrogenic to ischemic fistula was 58.5% versus 41.5%, respectively. Grossly, this does not highlight the alarming advancing rate of iatrogenic fistula. Thus, we wanted to determine the yearly etiological trends in urogenital fistula at a single center over several years. Our study found a rising trend in iatrogenic fistula: 43.5% (2006–08), 51.0% (2009–10), 57.8% (2011–12), 61.1% (2013–14), 68.4% (2015–16), 71.4% (2017–18). This overlooked rising trend in iatrogenic fistula is emerging as a new challenge to the health sector, particularly in low‐income countries.

The gradual rise in iatrogenic fistula is a warning sign about the quality of health care and training systems. Although surgical training offers a possible solution, health personnel may not have gained adequate practical experience to deal with complicated deliveries and surgical procedures. Therefore, there is a need for advanced training for improved decision‐making and surgical skills in both obstetric and gynecologic management, especially for safe cesarean delivery and hysterectomy. Furthermore, despite the declining trend in obstetric fistula, measures must also be taken to further improve health services in access‐restricted areas. These measures will ultimately lead to a better healthcare system and decrease the rate of fistula development.

In conclusion, the present study observed a rising trend in iatrogenic fistula over almost 12 years at a single center in Islamabad, Pakistan. These recent increasing trends in iatrogenic urogenital fistula emphasize the importance of improving safety standards for surgical techniques, both obstetric and gynecologic.

## AUTHOR CONTRIBUTIONS

NT designed and directed the project and drafted the manuscript. KB directed the project and carried out data collection. OA performed data analysis and interpretation and drafted and critically revised the manuscript. SL and HH conducted data collection.

## CONFLICTS OF INTEREST

The authors have no conflicts of interest.
